# Nidogen in development and disease

**DOI:** 10.3389/fcell.2024.1380542

**Published:** 2024-03-14

**Authors:** Uwe Töpfer, Anne Holz

**Affiliations:** ^1^ Department of Cellular and Physiological Sciences, University of British Columbia, Vancouver, BC, Canada; ^2^ Institut für Allgemeine und Spezielle Zoologie, Justus-Liebig-Universität Giessen, Giessen, Germany

**Keywords:** basement membrane, collagen, dandy-walker malformation, entactin, extracellular matrix, laminin

## Abstract

Nidogen, also known as entactin, is a multifunctional glycoprotein that plays a crucial role in the maintenance of the basement membrane (BM), morphogenesis and neuronal plasticity. This review aims to provide an overview of the structural features, molecular interactions and diverse functions associated with Nidogen. As a bridging molecule within the BM, Nidogen acts as a linchpin connecting various extracellular matrix (ECM) components. Its involvement in tissue development, homeostasis, and pathological conditions underscores its biological and medical significance. We discuss the current state of knowledge regarding Nidogen’s role in tissue maintenance, cell adhesion, migration, and signaling, shedding light on its intricate contributions to physiological and pathological processes.

## 1 Introduction

The basement membrane (BM) is a specialized extracellular matrix (ECM) with a thickness of 20–50 nm (Kefalides and Borel, 2005) that is crucial for tissue morphogenesis and homeostasis. It is responsible for maintaining tissue structural integrity and function (Feitosa et al., 2011; Jayadev and Sherwood, 2017; Sekiguchi and Yamada, 2018). Nidogen plays a crucial role in maintaining tissue homeostasis and dynamics by linking different extracellular matrix (ECM) components, affecting their molecular architecture and mechanical stability (Bader et al., 2005; Dai et al., 2018; Wolfstetter et al., 2019; Töpfer, 2023).

Nidogen occupies a unique niche within the BM, forming interactions with Laminins and other matrix constituents (Yurchenco, 2011). It is essential not only in embryonic development but also in the pathophysiology of various diseases. Humans have two *Nidogen* genes (*NID-1* and *NID-2*), while invertebrates encode a single *Nidogen* gene, (Ho et al., 2008; Shao et al., 2023). Human NID-1 and NID-2 share 46% homology on the amino acid level resulting in a strong structural conservation despite a low sequence homology (Erickson and Couchman, 2000; Miosge et al., 2000). Both isoforms are found in all BMs with a similar distribution during embryonic development while NID-2 is later restricted to some mature BMs (Miosge et al., 2000; Bader et al., 2005; Ho et al., 2008; Bechtel et al., 2012). Beyond its structural role, several studies have unveiled Nidogen´s participation in cell signaling, tissue development, and maintenance of organ homeostasis. Loss of Nidogen can lead to syndactyly, changes in limb development, and perinatal lethality, as well as abnormalities in lung development and cardiac tissue integrity (Bader et al., 2005; Böse et al., 2006). Nidogen-Integrin interaction may play a role in cell attachment, as evidenced by Nidogen-1 binding to αvβ3 and α3β1 dimers (Dedhar et al., 1992; Dong et al., 1995). Furthermore, a complex of Integrin, Nidogen, and Laminin has been proposed to regulate the stem cell niche and epidermal maintenance (Muffler et al., 2008). However, the molecular mechanisms responsible for Nidogen functions in terms of tissue integrity or disease are not yet fully understood.

This mini-review synthesizes the current state of knowledge surrounding Nidogen, shedding light on its structural domains, interaction partners, and functional implications across different biological contexts. By combining findings from disparate fields, we strive to provide a comprehensive overview that not only underscores the significance of Nidogen but also identifies knowledge gaps warranting further exploration. In doing so, we aim to inspire future investigations that will contribute to a better understanding of this multifaceted gene and its implications for development and disease.

## 2 Macromolecular functions

**FIGURE 1:**
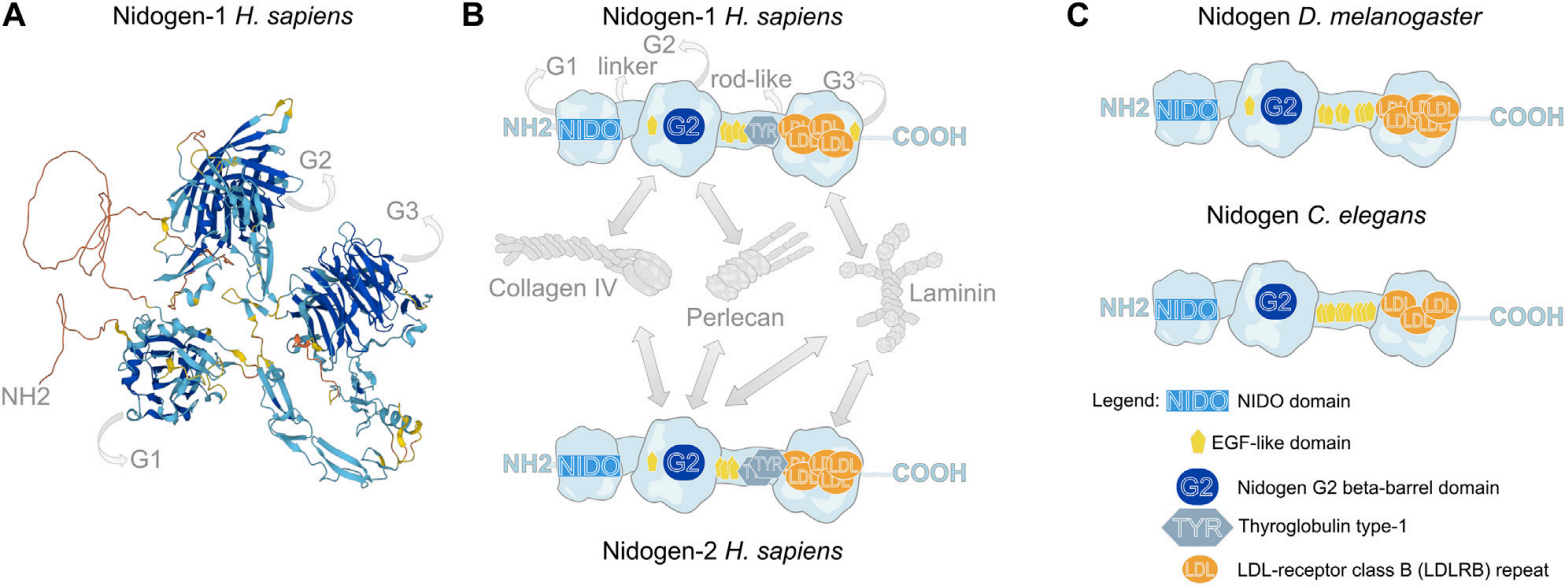
Molecular structure of Nidogen. **(A)** Alpha fold-based model of human Nidogen-1 structure. **(B)** Schematic illustration of the domain structure of human Nidogen-1 and Nidogen-2. Nidogen has three globular domains (G1-G3), which are separated by a short flexible linker region between G1 and G2 and a rod-like domain between G2 and G3. G1 contains a NIDO-domain, while the G2 domain comprises a Nidogen G2 beta-barrel domain and an EGF-like domain, the rod-like domain includes EGF-like domains as well as a Thyroglobulin type-1 domain and the G3 domain is mainly composed of LDL-receptor class B repeats. Interactions with Collagen IV, Perlecan and Laminin are shown with grey arrows. **(C)** The protein domain structure of *Drosophila* Nidogen and *C. elegans* Nidogen shows high similarities with only minor differences in the number of LDL repeats. Notably, invertebrates do not show Thyroglobulin type-1 domains. UniProt database was used for molecular and domain structures (Consortium, 2023).

Nidogen is classified as a linker protein because it interacts with multiple partners, such as Laminin, Collagen IV, Perlecan, and Integrin (Figure 1). Therefore, it has been attributed a significant role in BM assembly. Although it interacts with both layers of Laminins and Collagens, the cross-linking between these layers is not yet fully understood (Aumailley et al., 1993; Mayer et al., 1993; Mayer et al., 1995; Ho et al., 2008).

### 2.1 Biochemical structure and interactions

Nidogen is a sulfate glycoprotein that consists of three globular domains (G1-G3), a short linker region and a rod-like domain (Durkin et al., 1988; Fox et al., 1991; Mayer et al., 1995) (Figure 1A,B). The G1 domain contains a ∼180 amino acid residue NIDO domain, an extracellular domain with unknown function, found in Nidogen and other ECM and cell adhesion proteins. The G2 domain is characterized by a beta-barrel, that contains a large surface patch and the G3 domain contains low-density lipoprotein receptor repeats (LDL) (Durkin et al., 1988). This molecular composition of functional domains is highly conserved across the animal kingdom (Hynes, 2012; Fidler et al., 2017) (Figure 1B,C). While the globular domains only differ concerning the amount of LDL repeats and the presence of an EGF-like domain in G2, the invertebrates *Drosophila* and *C. elegans* do not have Thyroglobulin type-1 domains in their rod-like segments (Figure 1B,C). The globular domains are essential for the diverse protein interactions of Nidogen, which are shown schematically for human NID-1 and NID-2 (Ho et al., 2008; Bechtel et al., 2012) (Figure 1B). Using protein binding assays with recombinant Nidogen, Collagen IV and Laminin were identified as interaction partners with high affinities of the G2 domain to Collagen IV and the G3 domain to Laminin (Aumailley et al., 1989; Mann et al., 1989). Thereby, Nidogen interacts with the short arm of the Laminin γ-subunit (Gerl et al., 1991). Moreover, Nidogen was identified as a protein required to build stable ternary complexes and act as the bridge between Laminin and Collagen IV (Fox et al., 1991; Aumailley et al., 1993; Reinhardt et al., 1993). Additionally, the G2 domain mediates the binding of Nidogen to Perlecan (Reinhardt et al., 1993; Hopf et al., 2001). In *Drosophila*, these protein interactions have been tested *in vivo*. Dai et al. (Dai et al., 2018) generated GFP-tagged Nidogen proteins with deletions of each of the three globular domains and tested their fluorescence intensity in the fat body BM after knockdown of Laminin or Collagen IV, respectively. The authors demonstrated that localization of Nidogen directed by the G3 domain depends on Laminin, whereas localization through the G1 and G2 domains depends on Collagen IV.

### 2.2 Basement membrane assembly and stabilization

Studies in *Drosophila* indicate that Laminin is crucial for the incorporation of Nidogen into the BM in embryos, fat body and the egg chamber (Dai et al., 2018; Wolfstetter et al., 2019; Töpfer et al., 2022). Interestingly, although Nidogen requires Laminin for recruitment, studies in *Drosophila*, *C. elegans* and mice show that Collagen IV and Perlecan are capable of assembling independently of Nidogen into the BM (Kang and Kramer, 2000; Bader et al., 2005; Dai et al., 2018; Wolfstetter et al., 2019; Töpfer and Holz, 2020; Töpfer et al., 2022). Thus, the *ex vivo* experiments, which identified stable ternary complexes that depend on Nidogen, are not required for the overall architecture of the BM *in vivo*. The relevance of the *ex vivo* protein interactions between Nidogen, with Collagen IV and Perlecan for the BM assembly, maintenance and stability remain unanswered and despite the postulated role as a molecular bridge and connector of BM components Nidogen seems not essential for initial BM formation, at least in lower organisms.

**FIGURE 2:**
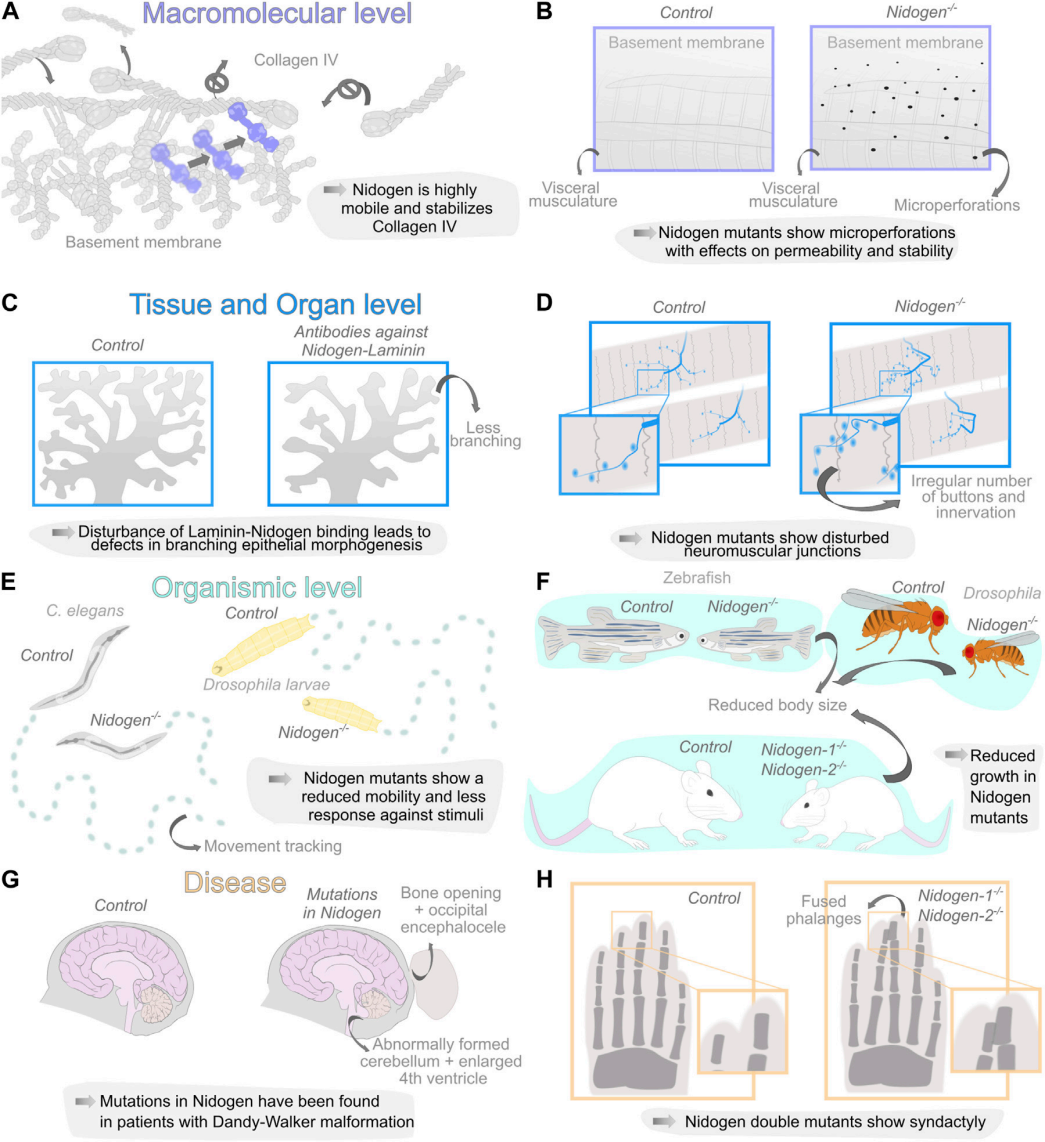
Diverse organization levels of Nidogen function during development and disease. **(A, B)** Nidogen has functions on the macromolecular level in linking the basement membrane (BM) components Laminin and Collagen IV due to the different protein domains. Loss of Nidogen in the *Drosophila* BM accelerates Collagen IV turnover **(A)** and results in a micro-perforated BM during embryonic development **(B)**. **(C, D)** Nidogen plays a crucial role on the level of tissue and organs. Nidogen loss results in branching defects during epithelial morphogenesis **(C)** and is necessary for maintaining structural integrity in neuromuscular junctions **(D)**. **(E, F)** The loss of Nidogen is associated with functions on the organismic level such as behavioral changes, including reduced mobility in *C. elegans* (left) and *D. melanogaster* larvae (right) **(E)** and size reduction in various organisms **(F)**. **(G, H)** The loss of *Nidogen* has correspondingly serious consequences for the development of various diseases such as Dandy-Walker malformation with occipital encephalocele **(G)** and soft tissue syndactyly of the extremities in mouse embryos of *Nidogen* double mutants were phalanges are joined together **(H)**.

Therefore, an interesting quantification approach of Collagen IV turnover revealed a highly dynamic nature of the BM during *Drosophila* embryonic development with a half-life of approximately 7 h. *Nidogen* mutants show a significantly higher turnover of Collagen IV during *Drosophila* embryogenesis, which implies that Nidogen prevents the degradation of Collagen IV (Matsubayashi et al., 2020) (Figure 2A) suggesting that Nidogen controls the stabilization of Collagen IV independently of the formation of a stable interaction. Interestingly, a comprehensive tagging of all relevant BM components in *C. elegans* (Keeley et al., 2020) revealed two classes of more or less dynamic proteins in the BM in Fluorescence Recovery After Photobleaching (FRAP) experiments. Nidogen is one of the highly dynamic proteins among Fibulin, Spondin and Agrin. Moreover, this experiment shows movement of Nidogen protein from unbleached into bleached BM areas, indicating high mobility of Nidogen in more static Laminin and Collagen IV scaffolds (Figure 2A). However, Nidogen turnover has not yet been quantified.

For a long time, studies examining the loss of Nidogen failed to detect abnormalities in the BM. Bader et al. were able to trace phenotypes in the lungs and hearts of mutant mice to corresponding BM defects in these organs (Bader et al., 2005). Using a GFP-tagged version of Collagen IV, a recent study also identified ruptures in the BM of the *Drosophila* fat body (Dai et al., 2018). Similarly, ultrastructural analysis using Scanning Electron Microscopy revealed changes in the BM of *Nidogen* mutants, with microperforations in the BM of the *Drosophila* larval visceral musculature (Wolfstetter et al., 2019) (Figure 2B). Indicating that Nidogen is vital for maintaining BM stability, which strengthens the BM and contributes to its overall structure and organization.

## 3 Tissue development and function

The crucial role for the formation and maintenance of tissues and organs is supported by the uncovering of molecular interactions in the fin fold BM of zebrafish (Zhang et al., 2022) and by the appearance of numerous defects at different levels of organization and in various tissues upon loss of Nidogen (Figure 2).

In kidney and lung epithelium, Nidogen is produced by adjacent mesenchymal cells (Miosge et al., 2000), indicating that mesenchymal Nidogen binding to epithelial Laminin may be a crucial event during epithelial development. Antibodies directed against the Nidogen-binding site on the Laminin B2 chain perturbed embryonic kidney and lung epithelial development *in vitro*. Thus, mesenchymal Nidogen could be important for early stages of epithelial morphogenesis (Ekblom et al., 1994). The analysis of branched epithelial morphogenesis of the submandibular glands in mice yielded similar findings. In this case, *Nidogen* mRNA was detected in the mesenchyme, while the protein was found in epithelial and endothelial BMs. This Nidogen-Laminin interaction was studied by applying antibodies to submandibular gland organ cultures. The antibodies interact with the Nidogen-binding site of the Laminin γ1 chain and drastically perturbed branching epithelial morphogenesis (Figure 2C), which leads to impaired BM formation between epithelium and mesenchyme (Kadoya et al., 1997).

On the other hand, epithelial BMs are very dynamic, as shown by strong remodeling processes during salivary gland branching in mouse development. There, the BM is perforated by hundreds of micro-holes in growth areas, resulting in a locally stretchable, mesh-like BM at the tips of the epithelial terminal buds necessary for their controlled epithelial expansion while maintaining tissue integrity (Harunaga et al., 2014; Töpfer, 2023). Also, breaching of BMs can be found during development when the anchor cell invades the BM of the vulva epithelium during vulva development in *C. elegans* and in vertebrates during the homeostasis of maturated tissues when immune cells pass the BM of endothelial cells (Sherwood and Sternberg, 2003; Hallmann et al., 2005; Glentis et al., 2014). These processes may only be made possible by the highly dynamic behaviour of Nidogen in BMs (Keeley et al., 2020). However, it remains questionable whether Nidogen behaves in the same dynamic manner in all types of BMs.

The role of Nidogen in supporting tissues mechanically has pronounced importance for neuronal development. In *C. elegans*, Nidogen is required for the positioning of longitudinal nerves by directing axon migration (Kim and Wadsworth, 2000) and the formation of neuromuscular junctions (Ackley et al., 2003). The authors found that Nidogen proteins accumulate laterally at the edge between muscle and nerves. The mutants showed mild movement phenotypes and a disturbed response against inhibitors of acetylcholinesterase and a cholinergic agonist. Therefore, the authors concluded that the function of the synapses is restricted (Ackley et al., 2003). Similar defects have been observed in *Drosophila* (Figure 2D,E). *Nidogen* mutants show a wide range of behavioral defects, such as reduced mobility, reduced reaction to vibrational stimuli, correct perception of gravitaxis, or climbing performance (Wolfstetter et al., 2019). The analysis of the larval nervous system morphology revealed improper positioning of dorsal dendritic arborization sensory neurons, aberrant chordotonal organ morphology and neuromuscular junction formation (Wolfstetter et al., 2019). In vertebrates, defects can already be observed in mutations of one of the two *Nidogen* genes. Studies on *Nidogen-1* mutants in mice have reported neurological phenotypes with seizure-like symptoms and loss of muscle control in the hind legs (Dong et al., 2002). On the other hand, Nidogen-2, which locally concentrates on synapses, is required for the structural maintenance of neuromuscular junctions in mice. While the neuromuscular junctions appear normal at birth, they show topological defects during maturation (Fox et al., 2008) arguing again for the role of Nidogen for maintaining BM stability in tissue morphogenesis.

This is also evident at the level of organ and individual development. *Nidogen* double mutant mice die a few days after birth due to defects in their lungs and hearts, specifically in the BMs of these organs (Bader et al., 2005). This indicates that Nidogen-1 and 2 are crucial for the proper formation and function of these BMs, which are essential for organ development and survival. It is important to note that not all BMs showed detectable alterations in the *Nidogen* double knockout mice, indicating that Nidogen-1 and 2 may have distinct roles in different tissues and that different BMs have different requirements for Nidogens (Bader et al., 2005). The fact that such changes in the BM are not found in all tissues, may indicate that structural abnormalities in the *Nidogen* mutants only become apparent when the tissue is subjected to high mechanical stress. Complementary, Willem et al., 2002 employed an elegant experimental approach by specifically ablating the Nidogen-binding sites in LamininC-1. Similar to the *Nidogen* double knockout situation, mice homozygous for the deletion die immediately after birth, exhibiting impaired lung development, renal agenesis, and locally restricted ruptures in the BM (Willem et al., 2002). It is reasonable to assume that these defects may be related to defects in epithelial morphogenesis in mice (Ekblom et al., 1994; Kadoya et al., 1997; Zhou et al., 2022). Nidogen mutants in invertebrates or double mutants in vertebrates are generally viable (Kang and Kramer, 2000; Böse et al., 2006; Dai et al., 2018; Wolfstetter et al., 2019) and show several fitness-related defects. Zebrafish *Nidogen* mutants exhibit a reduced average body length (Zhu et al., 2017). In addition, *Nidogen* double knockout mice are smaller than their wild-type littermates (Bader et al., 2005) and in *Drosophila*, a reduced size of *Nidogen* mutants was reported (Dai et al., 2018; Wolfstetter et al., 2019) (Figure 2F). Furthermore *C. elegans* and *Drosophila Nidogen* mutants show mildly reduced fecundity (Kang and Kramer, 2000; Dai et al., 2018; Wolfstetter et al., 2019).

The general viability of *Nidogen* mutants as well as their relative mild phenotypes raises the question of the gene’s importance and why Nidogen´s are highly conserved across the animal kingdom. In vertebrates, the compensation hypothesis, according to which NID-2 takes over functions of NID-1 in the BM, may explain the limited phenotypes observed after NID-1 elimination (Miosge et al., 2002). Despite the ubiquitous presence of Nidogen in BMs, corresponding defects were not found in all tissues even in invertebrates that do not have redundant Nidogen genes, suggesting different roles of Nidogen in different BMs and/or differently composed BMs. Therefore, together with the recently discovered motile behaviour of Nidogen in BMs, we propose that Nidogen acts as a pivotal point in transient protein interactions in BMs. Accordingly, phenotypes would be more likely found in BMs with low Nidogen mobility, while in BMs with a high Nidogen mobility the protein is more likely dispensable.

## 4 Nidogen in disease

Mutations in Nidogen have been linked to developmental disorders and human diseases due to its role in ensuring the structural integrity of BMs. This is especially true for the interaction of Nidogen with Laminin and the resulting defects during epithelial morphogenesis, which leads to dysregulated signaling events and numerous abnormalities during development (Figure 2G,H).

Several studies reveal Nidogen-Laminin interactions as basis for the important role for Nidogen-2 (NID-2) during cancer metastasis and NID-2 has been recently identified as a suitable biomarker for cancer progression (Chettiankandy et al., 2022). NID-2 is assumed to stabilize BMs by connecting Laminins to Collagen IV. Therefore, loss of NID-2 expression destabilizes BMs, leading to their degradation and increasing the invasive ability of epithelial cells. The loss of NID-2 is caused by aberrant hypermethylation of its promoters. Consequently, loss of NID-2 has been implicated in various malignant neoplasms, such as gastric and lung carcinomas (Guerrero-Preston et al., 2011; Chai et al., 2018; Srisuttee et al., 2020; Chettiankandy et al., 2022). Additionally, absence of NID-2 results in increased lung metastasis in mice (Mokkapati et al., 2012). This form of cancer events may be associated with the role of Nidogen in epithelial morphogenesis. While on the other hand, the events leading to dysregulation of NID-2 expression and thus loss of NID-2 are probably more likely to be related to signaling events such as BMP, EGF and FGF signaling that were also found during epithelial morphogenesis (Ekblom et al., 1994; Kadoya et al., 1997).

Interestingly, Nidogen-Integrin and Nidogen-Laminin interactions are also important for neurodegeneration. Lee et al. (Lee et al., 2007) demonstrate that Nidogen-1 serves two functions in adult Schwann cells, a specialized form of glia cell, which forms a sheath cell that envelops the axon of a peripheral neuron and, in the case of fibers containing marrow, is electrically insulated by a myelin sheath (Fallon and Tadi, 2024). Firstly, it increases Schwann cell formation, and secondly, it protects them from induced cell death. Both processes are likely dependent on the interaction of Nidogen-1 with ß1 Integrin. The authors speculate, that the prosurvival role could be affected by an overcompensation of other Integrins in a Nidogen-dependent change of ß1 Integrin level. Similarly, mice mutants for the Nidogen-1 interaction partner LamininC1 in Schwann cells display disrupted BM and peripheral nerve function (Chen and Strickland, 2003). A recent study found that Nidogen-1 was one of four proteins significantly reduced in patients with schizophrenia (Rodrigues-Amorim et al., 2022). The progressive neurodegeneration in schizophrenia is consistent with the previously discussed role of Nidogen-1 in maintaining BM structure and may indicate a prosurvival role of Nidogen-1 in Schwann cell development (Lee et al., 2009). This provides further evidence of ECM-dependent neuronal plasticity that can be disrupted by Nidogen-1 ablation, leading also to epileptic activity (Köhling et al., 2006; Vasudevan et al., 2010; Zhou et al., 2022).

Similarly, in a human disease Nidogen-Laminin interactions seem involved in expression of the illness. Case reports of patients with autosomal dominant Dandy-Walker malformation and occipital encephaloceles found mutations in *Nidogen1* and the Nidogen binding region of *LamininC1* (Darbro et al., 2013; Chai et al., 2018; McNiven et al., 2019; Dietvorst et al., 2023). The patients showed a broad spectrum of phenotypic variability including small bony defects, arachnoid cysts and occipital encephaloceles (Figure 2G). However, the causal relationship between this genetic mutation and the disease remains unclear, and multiple factors likely contribute to its progression. Interestingly, several studies of *Nidogen* mutants show phenotypes similar to the symptoms of Dandy-Walker malformation. First, Dong and co-workers (Dong et al., 2002) showed that deletions of *Nidogen-1* in mice exhibit capillary BM defects and behavioral changes. Thus, Nidogen seems to play a role in the maintenance of capillary integrity due to the caused BM defects (Marchand et al., 2019). Second, a former analysis of Dandy-Walker syndrome revealed that mutations in *Zic* and *Foxc1* cause ECM changes in the mesenchymal cells that are additionally responsible for the developmental pathogenesis of the cerebellum (Aldinger et al., 2009; Blank et al., 2011). Third, bone defects were detected in mouse limb development in *Nidogen* mutants (Figure 2H). While mice carrying at least one *Nidogen* allele exhibit normally formed limbs at birth, all *Nidogen-1/2* deficient mice exhibit cutaneous syndactyly of both fore and hind limbs. The lack of *Nidogen-1/2* results in defective formation of the ectodermal limb bud BM, which leads to aberrant apical ectodermal ridge formation. This in turn causes a changed distribution of growth factors required there and thus leads to a fully penetrant syndactyly of the soft tissue, where interdigital apoptosis is dysregulated (Böse et al., 2006). Which in turn demonstrates again the close connection between ECM-dependent epithelial morphogenesis and signaling events.

## 5 Conclusion and outlook

How Nidogen acts on the molecular level to ensure proper development of tissue and organs and their homeostasis remains largely unknown. Multidisciplinary approaches will be required to uncover how the biochemical interactions and the dynamic nature of Nidogen contribute to the biophysical properties of the BM. In particular, it is not yet clear how Nidogen, which moves rapidly through the Laminin and Collagen IV scaffolds, affects the stability of other components and the entire BM. It remains also questionable whether Nidogen behaves in the same dynamic manner in all types of BMs and whether these dynamics are regulated. Possibly, the binding affinity towards its interacting partners may be modulated by signaling molecules to modify the overall mechanical properties of the BM. The use of tissue-specific analyzes in combination with novel imaging techniques and genetic tools in model organisms can help to elucidate the complex spectrum of phenotypes present in various tissues. Understanding the role of Nidogen in BM assembly and stabilization can provide insight into the function of BMs in tissue morphogenesis and maintenance, as well as pathological conditions where Nidogen function might be disrupted, such as the Dandy-Walker malformation.
